# IL-17 sequestration via salivary gland gene therapy in a mouse model of Sjogren’s syndrome suppresses disease-associated expression of the putative autoantigen Klk1b22

**DOI:** 10.1186/s13075-015-0714-2

**Published:** 2015-08-06

**Authors:** Changgong Wu, Zhimin Wang, Lee Zourelias, Hiteshi Thakker, Michael J. Passineau

**Affiliations:** Gene Therapy Program, Department of Medicine, Division of Cardiovascular Medicine, Allegheny Health Network, Room 841, South Tower, 320 East North Avenue, Pittsburgh, PA 15212-4772 USA

## Abstract

**Introduction:**

IL-17 has a putative role in the pathophysiology of Sjogren’s syndrome (SS) and has been shown to be upregulated in the salivary glands of affected individuals. Sequestration of IL-17 with Adenoviral-mediated gene therapy has previously shown a benefit upon the SS-like phenotype in the Aec1/Aec2 mouse model. We sought to understand the proteomic consequences of IL-17 sequestration in the salivary gland of this mouse model as a means of illuminating the role of IL-17 in SS-like disease.

**Methods:**

Ultrasound-assisted gene transfer (UAGT) was utilized to express a fusion protein composed of the extracellular portion of the IL-17 receptor fused to fragment of crystallization (Fc) in the submandibular glands of Aec1/Aec2 mice at 8 weeks of age. After confirming expression of the fusion protein and local and systemic sequestration of IL-17, proteomic profiling was performed on submandibular glands of a treated cohort of Aec1/Aec2 animals relative to the background strain and sham-treated animals.

**Results:**

The most notable proteomic signatures of IL-17 sequestration on SS-like disease-related proteins were Kallikrein-related peptidases, including the putative autoantigen Klk1b22. IL-17 sequestration also notably led to an isoelectric shift, but not a molecular weight shift, of Kallikrein-1, attributed to phosphorylation.

**Conclusion:**

Non-viral IL-17 sequestration gene therapy in the salivary gland is feasible and downregulates expression of a putative SS autoantigen in the Aec1/Aec2 mouse.

**Electronic supplementary material:**

The online version of this article (doi:10.1186/s13075-015-0714-2) contains supplementary material, which is available to authorized users.

## Introduction

Sjogren’s syndrome (SS) is a systemic autoimmune disease affecting multiple organ systems and is the second most common rheumatic illness in the USA, affecting approximately 4 million Americans, 90 % of whom are female. The most common manifestations of SS are dry eyes and dry mouth, due to the characteristic exocrinopathy affecting the salivary and lacrimal glands. Despite the systemic comorbidities of SS, the disease has historically and practically been classified as a dry mouth/dry eye condition, leading the American College of Rheumatology to issue diagnostic guidelines focused on these two symptoms [1].

The molecular etiology of SS is poorly understood and appears to be very complex, based upon the findings that a variety of signaling pathways have been shown to be dysregulated in human salivary gland biopsies and animal models. SS appears to have a variable polygenetic basis [2], and thus patient-to-patient disease heterogeneity may explain the difficulty researchers have encountered in the search for a common cellular or molecular pathobiology in SS. At present, research efforts to unravel SS tend to focus on mechanisms underlying lymphocytic infiltration of the salivary gland, as such infiltration is pathognomonic and thus a reasonable candidate for an advanced deleterious state upon which multiple pathways converge.

In this research context, and in the absence of effective therapies for SS, gene therapy has been proposed as a means of disrupting the pathobiological cascade leading to salivary gland infiltration, dysfunction, and ultimately destruction. For such an approach to be successful, a gene drug capable of disrupting the pathobiology of SS at a convergence point proximal to lymphocytic infiltration of the salivary gland is required. Previous reports embodying this concept have utilized viral vector systems to deliver human vasoactive intestinal peptide [3], IL-27 [4], and cytotoxic T-lymphocyte antigen 4 [5], each showing beneficial effects upon Sjogren’s-like disease in animals models.

All previous demonstrations of gene therapy in animal models of SS have utilized a virus as the gene transfer vector. This approach is efficient and provides important proof of principle, but the clinical practicality of infusing a virus into the salivary gland to treat SS may not be optimal, as host immune response to the viral vector may contribute negatively to disease progression [6]. As an alternative, our group has developed and successfully demonstrated ultrasound-assisted gene transfer (UAGT) to the salivary gland using a combination of microbubbles and low frequency ultrasound that produces a sonoporation effect, allowing efficient entry of non-viral vectors into the cells of the salivary gland.

The present study sought to dissect the molecular basis of the impressive therapeutic effects of IL-17 sequestration reported in Nguyen et al. [7], who showed that sequestration of IL-17 by using Adenoviral gene transfer to express a fusion protein called IL-17R:Fc improves function and histological metrics in the Aec1/Aec2 model of SS [7]. This fusion protein is formed from the soluble portion of the IL-17 receptor fused to fragment crystallizable region (Fc), yielding a molecule that can efficiently bind and sequester free IL-17. This same group had earlier shown that adenoviral-mediated expression of IL-17A in healthy, non-SS-prone mice induces an SS-like disease [8]. IL-17 is a particularly interesting therapeutic target in SS both due to the putative role of IL-17 secreting T cells in the human disease, and the availability of emerging anti-IL-17 monoclonal antibodies as approved agents in other rheumatic diseases. Using UAGT to deliver the IL-17R:Fc cDNA to the Aec1/Aec2 mouse, we sought to compare the proteomic profiles of salivary gland tissue from IL-17R:Fc-treated animals, control animals treated with an irrelevant Luciferase (Luc) gene, and the background strain. Our hypothesis was that IL-17R:Fc gene therapy would shift the proteomic profile of the salivary gland of Aec1/Aec2 animals closer to that of the background strain, relative to Luc-treated animals.

## Methods

### Animals, husbandry, and experimental group design

The Institutional Animal Care and Use Committee of Allegheny Health Network Research Institute approved all animal experimentation described herein. The C57BL/6.NOD-Aec1 Aec2 mouse line [9] was acquired as a kind gift from Dr Ammon Peck (University of Florida, Gainesville, FL, USA) and mice were genotyped to confirm their identity using the probes: D3Mit151-F: 5′-GGTAAAATATTTTCTGGGCAAGC, D3Mit151-R: 5′-TTGTTAATTGTAATTCTGTTTCTGTCG. All gene transfer was performed on animals at 8 weeks of age.

A total of 48 mice were utilized in this study. One cohort of Aec1/Aec2 mice (n = 33) was assigned for determination of relative levels of IL-17R:Fc transcript and local and systemic levels of IL-17 protein. These animals received the following treatments: naïve (n = 2), UAGT/IL17R:Fc (n = 4), AdIL17R:Fc 1 × 10^7^ viral particles (vp) (n = 4), AdIL17R:Fc 1 × 10^8^ vp (n = 5), and AdIL17R:Fc 1 × 10^9^ vp (n = 5) and were sacrificed 48 hours after gene transfer. A second cohort (n = 15) was utilized for proteomic profiling of submandibular glands following gene transfer. These Aec1/Aec2 animals received the following treatments: UAGT/Luc (n = 5) and UAGT/IL17R:Fc (n = 5). A third group in this cohort (n = 5) were C57/BL6 background strain animals who received sham gene transfer consisting of anesthesia and ductal cannulation.

### Vector design and preparation

The plasmid vectors pCMV-GL3 (Luc) have been previously described by our group [10] and were used to generate the pCMV-IL17R:Fc vector, ensuring both vectors were isogenic with respect to the backbone. The adenoviral vector expressing IL-17R:Fc (AdIL17R:Fc) was acquired as a kind gift from Dr Jay Kolls (University of Pittsburgh, Pittsburgh, PA, USA) and was upscaled and purified using standard methods previously described by our group [11]. The cDNA for the IL-17R:Fc fusion protein was generated from adenoviral genomic DNA, sequence verified, and cloned into pCMV-GL3, replacing the GL3 sequence with the IL-17R:Fc sequence. Plasmid vectors were upscaled by growing in DH5α-competent cells (Life Technologies, Carlsbad, CA, USA) and vectors were purified using CsCl gradient ultracentrifugation.

### Salivary gland gene transfer

At 8 weeks of age, Aec1/Aec2 animals or background C57/BL6 animals received gene transfer to the submandibular glands bilaterally via UAGT or adenovirus. UAGT was performed as we have previously described [10]. Briefly, animals were anesthetized with a mixture of ketamine and xylazine and the submandibular duct was cannulated bilaterally, then 50 μl of solution containing either the adenoviral vector or 15 % v/v Definity microbubbles and 1 μg/μl of plasmid vector in normal saline was infused. Bubbles were destroyed by four 30-s bursts from a Sonigene device (Visualsonics, Inc., Toronto, ON, Canada) set for 1 MHz, 50 % duty cycle and 2 W/cm^2^, with 10 s between pulses. Following the four pulses, the transducer was withdrawn and the animal was rested for 10 minutes before the catheter was removed.

### IL-17R:Fc transcript and IL-17 protein detection in submandibular glands

At 48 hours after gene transfer, animals were sacrificed by cervical dislocation and submandibular glands removed and homogenized in RNALater. Total RNA was isolated from homogenized tissue using the RNEasy protocol (Qiagen, Venlo, Limburg Netherlands) and cDNA synthesized using a first strand synthesis kit (Roche Life Sciences, Indianapolis, Indiana USA). IL-17R:Fc transcript levels were determined by CYBR green quantitative real-time reverse transcription PCR using mouse glyceraldehyde 3-phosphate dehydrogenase (GAPDH) transcription level as an internal control on a Roche LightCycler® 480. The following primers were used: IF-17R:Fc-F: 5′-ATGGCTGCTTCTGCTGCT, IL-17R:Fc-R: 5′- CTTGACTCTGCAGCTCAGCC -3′ GAPDH-F: 5′- CGTCCCGTAGACAAAATGGT, GAPDH-R: 5′- TTGATGGCAACAATCTCCAC. The PCR program used was: 1) 95 °C, 5 minutes and 2) 95 °C 10 s, 60 °C 10 s, 72 °C 10 s, with 45 cycles.

An aliquot of these samples was analyzed by western blot. Protein concentrations were evaluated using the Bradford method and adjusting protein concentration to 1 μg/μl: 20 μg of each individual sample was separated by 15 % SDS-PAGE and transferred onto nitrocellulose membrane. The membranes were blocked with 5 % milk and probed with anti-IL-17 (1:2,000) (Abcam, Inc. Cambridge, MA, USA) and visualized using a horseradish-peroxidase-linked secondary antibody and Amersham ECL prime Western blotting Detection Reagent (GE Healthcare, Pittsburgh, PA, USA). GAPDH (Abcam) was used as a loading control.

### Serum IL-17 measurement

At 48 hours after gene transfer animals were sacrificed and blood collected by cardiac puncture. Serum IL-17 concentrations were determined by the Bio-Plex Pro™ Assay (Bio-Rad, Hercules, CA, USA).

### Proteomic profiling of salivary glands

Animals were sacrificed 21 days after gene transfer (to mimic the optimal functional improvements observed by Nguyen et al. [7]) by cervical dislocation and submandibular glands removed and homogenized on ice using 2D lysis buffer (7 M Urea, 2 M Thiourea, 2 % CHAPS). Samples were cleaned using a 2-D Quant kit (GE Healthcare) and resuspended in 2D gel rehydration buffer (7 M Urea, 2 M Thiourea, 2 % CHAPS) containing 50 mM Tris pH8.5. Protein concentrations were determined using the Bradford method and adjusted to 1 μg/μl. Profiling was performed initially on pooled samples (n = animals/group): 1 μl of Cy2, Cy3 or Cy5 were added to 50 μg of each pooled group sample respectively. Three gels were performed with dye swapping, to ensure that each pooled group was labeled with each Cy dye. After addition of dye, samples were incubated on ice for 30 minutes in the dark. After 30 minutes, 1 μl of 10 mM lysine was used to quench the reaction by incubating on ice for 10 minutes in the dark.

Isoelectric focusing (IEF) and SDS electrophoresis were performed as we have previously described [12]. Briefly, 15 μg of each labeled sample was diluted in rehydration buffer to 450 μl for IEF. A 24-cm strip, pH3-10NL was rehydrated by adding 2 % DTT, 0.5 % IPG buffer and 0.002 % bromophenol blue room temperature for 8 hours. Samples were applied to rehydrated strips and loaded onto an Ettan IPGphor 3. IEF was performed overnight for 60,000 volt-hours (vhr). The next day, the strips were equilibrated with 1 % DTT followed by 2.5 % iodoacetamide for 15 minutes each and separated in a 13.5 % SDS gel. Finished gels were scanned on a Typhoon Trio (GE Healthcare). The pictures were edited using ImageQuant TL 7.0 software (GE Healthcare). Differential in-gel analysis (DIA) and biological variation analysis (BVA) of the two-dimensional differential gel electrophoresis (2D DIGE) results were performed using DeCyder 2D 7.0 Software (GE Healthcare).

### Pairwise comparisons

In order to confirm the statistical validity of spots of interest identified by DIA analysis, pairwise comparisons of the various groups were made to allow BVA. Pairwise comparisons were performed on randomly paired individual samples from each group labeled respectively with Cy3 or Cy5 or vice versa and using a mixed sample labeled using Cy2 as an internal standard.

### Phosophoprotein staining

In order to determine the phosphorylation of spots 4 and 5, 2-D gels were run (as described above) with a single sample, either UAGT/Luc and UAGT/IL-17R:Fc, and stained with Pro-Q Diamond phosphoprotein gel stain reagent for phosphorylated protein. The locations of spots 4 and 5 were identified using landmarks, and the gels were washed and stained with Sypro Ruby for total protein. Spots 4 and 5 were then cut from the gel and protein ID performed to confirm their identity. Both reagents were obtained from Thermo Fisher Scientific Inc. Waltham, MA USA.

### Protein identification

Protein identification was performed as previously described [13]. Briefly, protein spots of interest were excised from the 2-D gel, reduced by DTT, alkylated with iodoacetamide, digested with trypsin and desalted with C18 ZipTips (Millipore Corporation, Billerica, MA, USA). Both mass spectrometry (MS) and tandem mass spectrometry (MS/MS) analyses of the digested peptides were performed on the matrix-assisted laser desorption/ionization time-of-flight mass spectrometry (MALDI-TOF)/TOF tandem MS (Bruker UltrafleXtreme MALDI TOF/TOF Mass Spectrometer, Bruker Daltonics Inc. Billerica, MA, USA). The database search and analysis were performed using FlexAnalysis and BioTools software (Bruker Daltonics Inc.) against mouse Swiss-Prot protein database using a local Mascot search engine.

### Statistical analysis

**Fig. 1:**
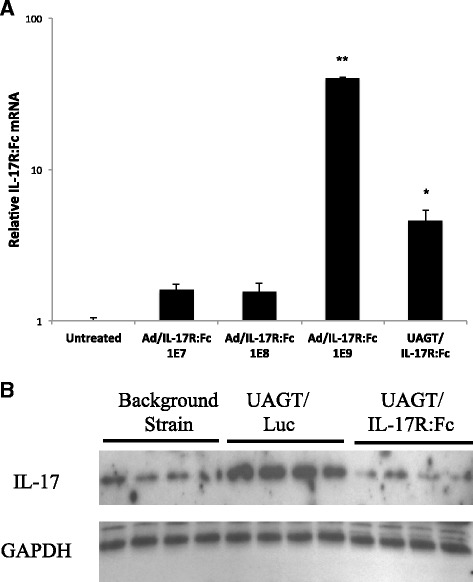
Local IL-17 sequestration following IL-17R:Fc gene transfer to the salivary gland of Aec1/Aec2 mice. **a** Relative mRNA levels of the IL-17R:Fc fusion protein following gene transfer with varying amounts of an adenovirus vector (1E7, n = 4, 1E8, n = 5, 1E9, n = 5) or ultrasound-assisted gene transfer (*UAGT*)/IL-17R:Fc (n = 4). Error bars are + standard error of the mean. **b** Western blot analysis of salivary gland tissue treated with UAGT/IL-17R:Fc relative to UAGT/Luciferase (*Luc*) and the background strain. Glyceraldehyde 3-phosphate dehydrogenase (*GAPDH*) was used as a housekeeping control

**Fig. 2:**
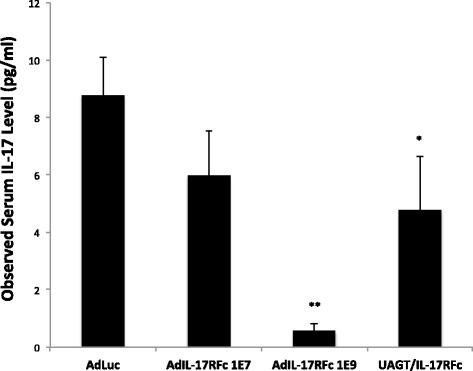
Relative serum levels of IL-17 in Aec1/Aec2 animals treated with gene transfer to the salivary glands. Plasma was collected from animals via cardiac puncture 48 hours after gene transfer to the submandibular glands. Dosages of adenoviral vector are expressed as viral particles/gland (1E7, n = 11, 1E9, n = 4). Error bars are + standard error of the mean. *Significant difference (*p* <0.05) between negative control (*AdLuc*, n = 9) and ultrasound-assisted gene transfer (*UAGT*)/IL-17R:Fc (n = 10). **Significant difference between 1E9 AdIL-17R:Fc and both AdLuc and UAGT/IL-17R:Fc

Group comparisons in Figs. 1a and 2 were made using the Mann-Whitney *U* test. Analysis of proteomic profiles was performed within the DeCyder software, with 2-fold changes as the threshold. DIA was used for pooled gels and BVA was used for pairwise comparisons. In all cases, statistical significance was considered to be *p* <0.05.

## Results

### Ultrasound-assisted gene transfer of the IL-17R:Fc cDNA results in expression levels of the fusion protein comparable to previously reported doses of adenovirus and reduces IL-17 protein to background strain levels

IL-17R:Fc expression from 1 × 10^7^ vp of Adenovirus has previously been reported to be sufficient for gene therapy efficacy in the Aec1/Aec2 mouse model [7]. We first sought to compare IL-17R:Fc mRNA expression levels after UAGT relative to the 1 × 10^7^ dose as well as the higher doses (1 × 10^9^−1 × 10^10^vp) that have been more commonly reported in the salivary gland gene therapy literature. Figure 1a shows relative mRNA levels from Aec1/Aec2 mouse submandibular glands 48 hours after gene transfer of IL-17R:Fc delivered by UAGT, 1 × 10^7^ vp, and 1 × 10^9^ vp of adenovirus. AdLuc gene transfer in the Aec1/Aec2 mice served as a negative control.

In order to demonstrate the functionality of IL-17R:Fc for reducing IL-17 protein in salivary gland tissue, we performed western blot analysis of salivary gland tissue 48 hours after UAGT/IL-17R:Fc, with UAGT/Luc serving as a negative control and the background strain serving as a positive control (Fig. 1b). These experiments demonstrated that IL-17 is highly expressed in the salivary glands of the Aec1/Aec2 mouse relative to the background strain, and that this increase is dramatically reversed by IL-17R:Fc gene transfer.

### IL-17R:Fc expression in the salivary gland of Aec1/Aec2 mice at 8 weeks of age significantly lowers serum IL-17

As an additional assurance that IL-17 levels were reduced by our gene therapy, we assayed serum levels of IL-17. Nguyen et al. previously reported that IL-17R:Fc gene therapy significantly reduces IL-17 levels systemically, although it is unknown whether this is due solely to local sequestration of IL-17 in the salivary gland, or whether IL-17R:Fc is secreted into the blood from the salivary gland. Blood was collected from the same animals studied in Fig. 1a and b and relative serum IL-17 levels were assayed, with results shown in Fig. 2. These results confirmed that our IL-17R:Fc was working as intended, and allowed us to move forward into proteomic profiling of the therapeutic effect.

### IL-17R:Fc expression in the salivary glands of Aec1/Aec2 mice suppresses expression and/or post-translational modification of disease-associated proteins, including the putative autoantigen Klk1b22

**Fig. 3:**
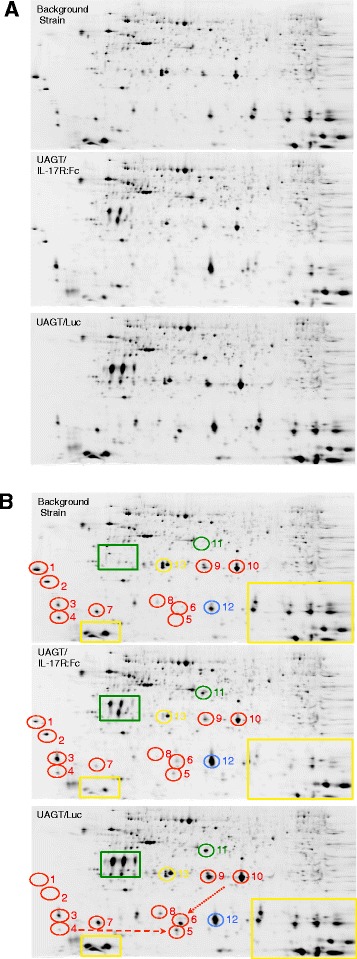
Proteomic profiling of mouse submandibular glands 21 days after gene transfer. **a** Fluorescent intensity map generated as an average of three gels, with rotation of Cy2, Cy3, and Cy5 dyes between the three pooled samples (n = 5/group). **b** Annotation of the intensity map shown in 3a. *UAGT* ultrasound-assisted gene transfer, *Luc* Luciferase

We performed difference gel electrophoresis on pooled samples of submandibular gland lysates from the three treatment groups (UAGT/IL-17R:Fc, UAGT/Luc, and background strain) 21 days after treatment, running all three pooled samples on the same gels (three gels were performed, to allow for swapping of the Cy dyes between groups) to generate aligned differential proteomic profiles as shown in Fig. 3a, and annotated in Fig. 3b. This analysis design allowed us to parse out the proteomic profile of the Aec1/Aec2 SS-like disease process (by comparing background strain to UAGT/Luc-treated), then to evaluate the effect of IL-17R:Fc treatment upon this process (by evaluating the effect of UAGT/IL17R:Fc treatment upon this dataset). Bioinformatic analysis, and post-hoc pairwise comparisons, as described in Methods, was undertaken to evaluate the statistical power of spots shown to be differentially expressed between the treatment groups.

They key findings of our proteomic analysis are annotated in Fig. 3b and summarized in Table 1. These proteins met the dual criteria of 1) being disease-associated and 2) being modulated toward background strain expression levels by IL-17R:Fc, and were thus designated as putative therapeutic targets of IL-17R:Fc gene therapy treatment. Suppression of local IL-17 expression was verified at the 21 days post-treatment time point (see Figure S1 in Additional file 1).

**Table 1:** Protein identities of spots meeting the criteria of being associated with SS-like disease in the Aec1/Aec2 model versus background strain and being modified toward background strain levels as a result of IL-17R:Fc gene therapy

Spot number	Protein identity	Peak volume		
		background strain	UAGT/Luc	UAGT/IL-17R:Fc
Disease-associated proteins, putative therapeutic targets of IL-17R:Fc (RED)				
1	Unidentified	80578	109	15102
2	E3 Ubiquitin protein ligase	62098	265	31004
3	Calmodulin	42723	18754	45846
4	Kallikrein-1, pl~3.8	26302	3034	11464
5	Kallikrein-1, pl~5.7	1050	13914	3664
6	Klk1b22 (fragment)	5973	34770	3056
7	Klk1b3 (nerve growth factor gamma)	32176	58767	3995
8	Klk1b16	9091	22805	2291
9	Klk1b22, pl~6.2	43192	153120	10928
10	Klk1b22, pl~6.7	180273	343252	190954

*SS* Sjrogen’s syndrome, *UAGT* ultrasound-assisted gene transfer, *Luc* Luciferase

**Fig. 4:**
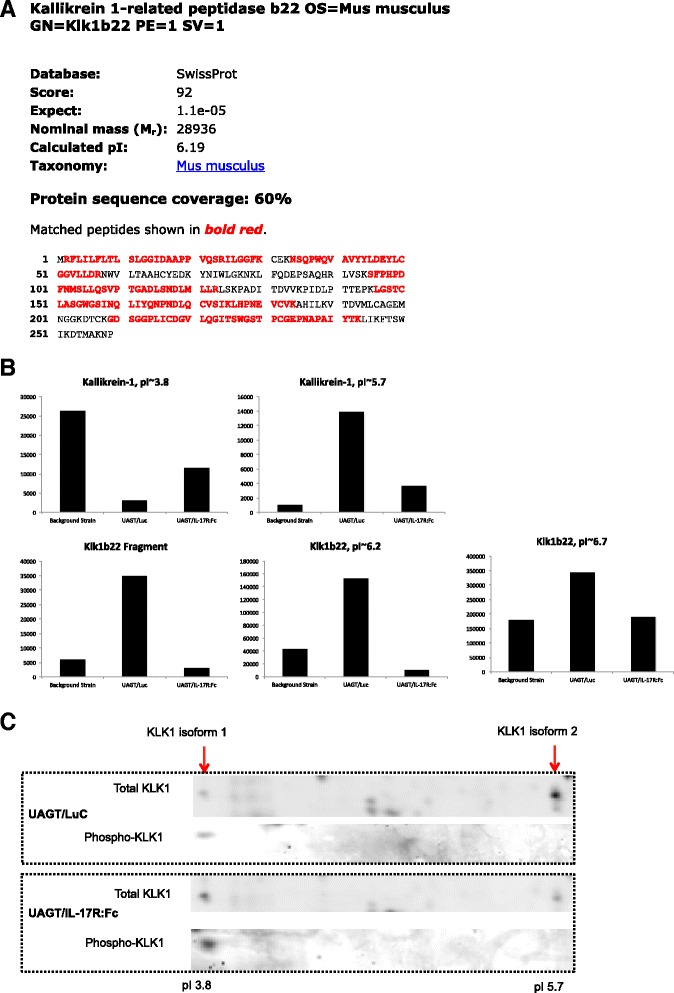
Identification and relative quantification of the putative biomarkers Kallikrein-1 and Klk1b22 after treatment with IL-17R:Fc gene therapy. **a** Mass spectrometry sequence coverage of spots 6, 9 and 10 identified as Klk1b22. **b** Gel imaging shown in Fig. 3 was subjected to relative quantitative analysis to measure fluorescent intensity of spots positively identified to be Kallikrein-1 (4 and 5) or Klk1b22 (6, 9, and 10). Numerical values of these intensities are shown in Table 1. **c** Magnification of 2-D gels run with either ultrasound-assisted gene transfer/Luciferase (*UAGT/Luc*) or UAGT/IL-17R:Fc samples, showing total Kallikrein-1 (KLK1, *top bars*) and phosphorylated Kallikrein-1 (*bottom bars*). Acidic shift of KLK1 is attributed to phosphorylation

Most notably, Il-17R:Fc gene therapy dramatically reduced expression of Klk1b22, which has been shown to be sufficient to cause keratoconjunctivitis sicca (KCS) when used to inoculate healthy Lewis rats [14]. Because a reliable antibody is not available for detecting mouse Klk1b22, we have provided sequence coverage obtained from spots 9 and 10 in Fig. 4a, demonstrating that we can make this identification with high confidence. Relative levels of Klk1b22 and Kallikrein-1 isoforms in the salivary glands of the background strain and treatment groups are illustrated graphically in Fig. 4b. Kallikrein-1 has been implicated as an autoantigen in the IQI/Jic mouse model of SS [15], and earlier studies associated sialylation of tissue Kallikreins with autoimmune diseases [16, 17]. We suspected that the isoelectric shift of Kallikrein-1 might be attributable to phosphorylation and Fig. 4c shows phosphoprotein staining of spots 4 and 5 with UAGT/Luc versus IL-17R:Fc treatment, strongly suggesting that IL-17R:Fc gene therapy enhances the phosphorylation state of Kallikrein-1, although not to background strain levels.

### IL-17R:Fc expression in the salivary glands of Aec1/Aec2 mice broadly suppresses expression of Kallikrein1-related peptidases

The proteome of the submandibular gland of the Aec1/Aec2 mouse, and that of the C57/BL6 background strain upon which it is based, is characterized by strong expression of highly basic, low molecular weight Kallikrein 1-related peptidases (yellow boxes in Fig. 3b, and Table 2). These peptidases are not associated with the SS-like disease in the Aec1/Aec2 mouse, but are broadly downregulated by IL-17R:Fc treatment. Taken together with the preceding results, it appears that the effects of IL-17 sequestration on Klk1-related peptidases are relatively unspecific. Table S1 (in Additional file 2), catalogs all proteins identified to be significantly different between UAGT/Luc and UAGT/IL-17R:Fc-treated salivary glands.

**Table 2:** Protein identities of spots of interest that did not meet our criteria for disease/therapy-associated biomarkers

Spot number	Protein identity
Proteins modulated by gene transfer (GREEN)	
Green box	Renin 1,2 unidentified
11	Renin 1,2
Disease-associated proteins unaffected by IL-17R:Fc (BLUE)	
12	Klk1b6/b26
Proteins unassociated with disease, but modulated by IL-17R:Fc (YELLOW)	
Small box	Klk1b16, Hemoglobin subunit beta 1, unidentified
Large box	Klk1b6/b26, Klk1b8/b26, Klk1b21/b24, Klk1b8/b16/b26, Hemoglobin subunit alpha, Klk1b9, Klk1b3/b6/b9/b11
13	Klk1b5

## Discussion

The presence of IL-17-expressing T cells, both CD4+/CD8− and CD4−/CD8− [18], in the salivary glands is strongly associated with SS in human biopsies and animal models [19–22]. These clinical findings amplify the translational potential of IL-17-blocking gene therapy and the potential relevance of the Aec1/Aec2 mouse model to understanding the molecular choreography of SS. With evidence accumulating that local expression of IL-17 in the salivary gland plays a detrimental role in the initiation and/or progression of SS [23], further focus on IL-17 sequestration, either through conventional pharmacotherapy (e.g., anti-IL-17 monoclonal antibodies) or gene therapy (e.g., IL-17R:Fc), appears promising.

Nguyen et al. [7] previously showed impressive therapeutic effects of IL-17R:Fc gene therapy in the Aec1/Aec2 mouse model, including functional, histological, and serological metrics of benefit. Our study adapted UAGT, a safe and clinically promising method of delivering IL-17R:Fc gene therapy without the immunogenic consequences of the adenoviral vector. By using this immunologically compatible approach, we can parse out the molecular consequences of IL-17R:Fc gene therapy in the salivary glands of this animal model, using the non-disease-prone background strain as an additional means of inferring putative disease relevance of proteins affected by IL-17 and its inhibition.

Our results provide additional momentum for the idea that IL-17R:Fc gene therapy may be a viable means of inhibiting IL-17 locally within the salivary gland. We achieved IL-17R:Fc expression levels equal to or greater than those needed to achieve therapeutic efficacy, without the need for a viral vector. We did not explore the duration of transgene expression in great detail, but have previously reported that UAGT drives transgene expression for several weeks in the salivary gland of mice [10] and have recently observed similar results in mini-swine [24]. This technique is minimally invasive and should be repeatable on an outpatient basis if extended therapy is required.

Our finding that IL-17 inhibition is most strongly associated with suppression of Klk1b22, a Kallikrein 1-related peptidase, is novel and may provide clues into the propagation of SS in salivary glands where IL-17-producing cells are resident. Little is known about the function of Klk1b22, and while it was originally believed to be an EGF-binding protein (EGF-BP) [25, 26], later studies demonstrated a lack of EGF binding and rather an ability to cleave Nerve Growth Factor [27]. It is therefore possible, although unproven, that Klk1b22 and Klk1b3 are related in this model, as Klk1b3 is NGF-gamma. Very recently, Jiang et al. [14] showed that Klk1b22, expressed as a recombinant protein, can induce SS-like experimental autoimmune KCS in otherwise healthy Lewis rats, a strain of animals not prone to SS-like disease. This followed an earlier report that the tissue kallikrein Klk-13 acts as an autoantigen to stimulate SS-like disease in the IQI/Jic mouse model [15].

Our study provides further evidence that Klk1b22 may be a molecular antigen responsible for the well-established phenomenon of inducible SS-like disease, where immunization of healthy animals with salivary gland or lacrimal gland extracts leads to the development of KCS [28–30]. Notably, the gene for Klk1b22 is on Chromosome 7, and thus not directly related to the Aec1 (Chromosome 3) and Aec2 (Chromosome 1) alleles responsible for SS-like disease in this animal model. Gene expression of the developing lacrimal gland in this mouse model has been characterized and includes differential expression of tissue kallikreins although Kallikrein-related peptidases were not mentioned [31].

To our knowledge, this is the first study suggesting a causal link between local IL-17 in the salivary gland and tissue levels of Klk1b22. While the expression levels of numerous molecules have been shown to be induced by IL-17, Kallikreins and Kallikrein-related peptidases are not among the known IL-17 inducible molecules although a recent study suggested an expanded range of gene expression downstream of IL-17 in keratinocytes that includes Klk1b6, Klk1b10, and Klk1b13 [32]. Our results suggest a causal link between IL-17 and tissue levels of Klk1b22, but the proximity of the cause and effect is as yet unknown, and could be remote. If Klk1b22 expression is indeed an autoantigen capable of inducing KCS, and this mechanism has any relevance to human SS, IL-17 could represent a therapeutic target upstream of irreversible glandular damage.

Many questions remain unanswered by our limited study. Beyond the obvious need for vetting the relevance of Klk1b22 to human SS, key questions to be addressed in future studies are 1) whether IL-17 sequestration can reverse Klk1b22 expression that is already manifest, and 2) whether systemic antagonism of IL-17, such as with the newer monoclonal antibodies Secukinumab and Izekizumab, leads to the same local suppression of Klk1b22 in the salivary gland, and 3) whether IL-17 blockade ablates production of anti-Klk1b22 autoantibodies. Given the indolent and progressive course of SS, there is some reason for hope that the window for blocking the biochemical cascade leading to tissue destruction may be wide.

## Conclusions

Non-viral IL-17 sequestration gene therapy in the salivary gland is feasible and downregulates expression of a putative SS autoantigen in the Aec1/Aec2 mouse. Based upon our results, further exploration of the therapeutic potential of IL-17 sequestration in Sjogren’s syndrome, either through gene therapy or biopharmaceuticals, is warranted.

## Additional files

Additional file 1: Figure S1. Western blot analysis of salivary gland tissue treated with UAGT/IL-17R:Fc relative to UAGT/Luciferase (Luc) and the background strain at 21 days post-treament. Actin was used as a housekeeping control. (PDF 69 kb) Additional file 2: Table S1. Proteins identified to be significantly different between UAGT/Luc and UAGT/IL-17R:Fc-treated salivary glands. (PDF 67 kb)

## Abbreviations

2D-DIGE: two-dimensional differential gel electrophoresis
Ad: adenovirus
BVA: biological variation analysis
CsCL: cesium chloride
DIA: differential in-gel analysis
EGF: epidermal growth factor
Fc: fragment of crystallization
GAPDH: glyceraldehyde 3-phosphate dehydrogenase
ID: identity
IEF: isoelectric focusing
IL: interleukin
KCS: keratoconjunctivitis Sicca
Luc: luciferase (also referred to as GL3)
MALDI-TOF: matrix-assisted laser desorption/ionization time-of-flight mass spectrometry
MS: mass spectrometry
MS/MS: tandem mass spectrometry
SDS: sodium dodecyl sulfate
SS: Sjogren’s syndrome
UAGT: ultrasound-assisted gene transfer
vp: viral particles
