# Supplementary figures and images for: IL-17 sequestration via salivary gland gene therapy in a mouse model of Sjogren’s syndrome suppresses disease-associated expression of the putative autoantigen Klk1b22
Source: Arthritis Res Ther. 2015 Aug 6;17(1):198. doi: 10.1186/s13075-015-0714-2 (PMC4527205; doi:10.1186/s13075-015-0714-2)

Background

UAGT/Luc

UAGT/IL-17R:Fc

IL-17

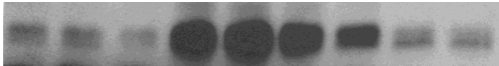

Actin

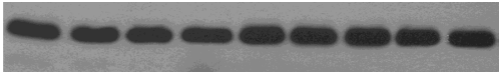

Supplement: Additional file 1: Figure S1. — Western blot analysis of salivary gland tissue treated with UAGT/IL-17R:Fc relative to UAGT/Luciferase (Luc) and the background strain at 21 days post-treament. Actin was used as a housekeeping control. (PDF 69 kb) [file 13075_2015_714_MOESM1_ESM.pdf]
