# Supplementary material for: IL-17 sequestration via salivary gland gene therapy in a mouse model of Sjogren’s syndrome suppresses disease-associated expression of the putative autoantigen Klk1b22
Source: Arthritis Res Ther. 2015 Aug 6;17(1):198. doi: 10.1186/s13075-015-0714-2 (PMC4527205; doi:10.1186/s13075-015-0714-2)
Supplement: Additional file 2: Table S1. — Proteins identified to be significantly different between UAGT/Luc and UAGT/IL-17R:Fc-treated salivary glands. (PDF 67 kb) [file 13075_2015_714_MOESM2_ESM.pdf]

Accession	Description	Downregulate in UAGT/IL-17R:Fc vs. UAGT/Luc	Upregulate in UAGT/IL-17R:Fc vs. UAGT/Luc
CH60_MOUSE	60 kDa heat shock protein, mitochondrial OS=Mus musculus GN=Hspd1 PE=1 SV=1		Y
RLA1_MOUSE	60S acidic ribosomal protein P1 OS=Mus musculus GN=Rplp1 PE=2 SV=1	Y	
GRP78_MOUSE	78 kDa glucose-regulated protein OS=Mus musculus GN=Hspa5 PE=1 SV=3		Y
CHIA_MOUSE	Acidic mammalian chitinase OS=Mus musculus GN=Chia PE=1 SV=2		Y
ACTC_MOUSE	Actin, alpha cardiac muscle 1 OS=Mus musculus GN=Actc1 PE=1 SV=1		Y
ACTS_MOUSE	Actin, alpha skeletal muscle OS=Mus musculus GN=Acta1 PE=1 SV=1		Y
ACTB_MOUSE	Actin, cytoplasmic 1 OS=Mus musculus GN=Actb PE=1 SV=1		Y
ACTG_MOUSE	Actin, cytoplasmic 2 OS=Mus musculus GN=Actg1 PE=1 SV=1		Y
ACTH_MOUSE	Actin, gamma-enteric smooth muscle OS=Mus musculus GN=Actg2 PE=1 SV=1		Y
ABHEB_MOUSE	Alpha/beta hydrolase domain-containing protein 14B OS=Mus musculus GN=Abhd14b PE=2 SV=1		Y
AMY1_MOUSE	Alpha-amylase 1 OS=Mus musculus GN=Amy1 PE=1 SV=2		Y
APOA1_MOUSE	Apolipoprotein A-I OS=Mus musculus GN=Apoa1 PE=1 SV=2		Y
ATPA_MOUSE	ATP synthase subunit alpha, mitochondrial OS=Mus musculus GN=Atp5a1 PE=1 SV=1		Y
ATPB_MOUSE	ATP synthase subunit beta, mitochondrial OS=Mus musculus GN=Atp5b PE=1 SV=2		Y
ATPG_MOUSE	ATP synthase subunit gamma, mitochondrial OS=Mus musculus GN=Atp5c1 PE=1 SV=1	Y	
IDLC_MOUSE	Axonemal dynein light intermediate polypeptide 1 OS=Mus musculus GN=Dnali1 PE=2 SV=1		Y
BPIA2_MOUSE	BPI fold-containing family A member 2 OS=Mus musculus GN=Bpifa2 PE=2 SV=1		Y
CALM_MOUSE	Calmodulin OS=Mus musculus GN=Calm1 PE=1 SV=2	Y	
CALR_MOUSE	Calreticulin OS=Mus musculus GN=Calr PE=1 SV=1		Y
CAH3_MOUSE	Carbonic anhydrase 3 OS=Mus musculus GN=Ca3 PE=1 SV=3		Y
CATB_MOUSE	Cathepsin B OS=Mus musculus GN=Ctsb PE=1 SV=2		Y
CISY_MOUSE	Citrate synthase, mitochondrial OS=Mus musculus GN=Cs PE=1 SV=1		Y
CO3_MOUSE	Complement C3 OS=Mus musculus GN=C3 PE=1 SV=3		Y
KCRU_MOUSE	Creatine kinase U-type, mitochondrial OS=Mus musculus GN=Ckmt1 PE=1 SV=1		Y
QCR2_MOUSE	Cytochrome b-c1 complex subunit 2, mitochondrial OS=Mus musculus GN=Uqcrc2 PE=1 SV=1		Y
RN187_MOUSE	E3 ubiquitin-protein ligase RNF187 OS=Mus musculus GN=Rnf187 PE=1 SV=2	Y	
ETFB_MOUSE	Electron transfer flavoprotein subunit beta OS=Mus musculus GN=Etfb PE=1 SV=3		Y
EF1A1_MOUSE	Elongation factor 1-alpha 1 OS=Mus musculus GN=Eef1a1 PE=1 SV=3	Y	
ENPL_MOUSE	Endoplasmin OS=Mus musculus GN=Hsp90b1 PE=1 SV=2	Y	
ECHM_MOUSE	Enoyl-CoA hydratase, mitochondrial OS=Mus musculus GN=Echs1 PE=1 SV=1	Y	
EGFB2_MOUSE	Epidermal growth factor-binding protein type B OS=Mus musculus GN=Egfbp2 PE=1 SV=1	Y	
ES1_MOUSE	ES1 protein homolog, mitochondrial OS=Mus musculus GN=D10Jhu81e PE=1 SV=1		Y
FRIL1_MOUSE	Ferritin light chain 1 OS=Mus musculus GN=Ftl1 PE=1 SV=2		Y
GLNA_MOUSE	Glutamine synthetase OS=Mus musculus GN=Glul PE=1 SV=6		Y
GPX1_MOUSE	Glutathione peroxidase 1 OS=Mus musculus GN=Gpx1 PE=1 SV=2		Y
GSTM1_MOUSE	Glutathione S-transferase Mu 1 OS=Mus musculus GN=Gstm1 PE=1 SV=2		Y
GSTM7_MOUSE	Glutathione S-transferase Mu 7 OS=Mus musculus GN=Gstm7 PE=1 SV=1		Y
G3P_MOUSE	Glyceraldehyde-3-phosphate dehydrogenase OS=Mus musculus GN=Gapdh PE=1 SV=2	Y	
GPDA_MOUSE	Glycerol-3-phosphate dehydrogenase [NAD(+)], cytoplasmic OS=Mus musculus GN=Gpd1 PE=1 SV=3		Y
GBLP_MOUSE	Guanine nucleotide-binding protein subunit beta-2-like 1 OS=Mus musculus GN=Gnb2l1 PE=1 SV=3		Y
HBA_MOUSE	Hemoglobin subunit alpha OS=Mus musculus GN=Hba PE=1 SV=2		Y
HBB1_MOUSE	Hemoglobin subunit beta-1 OS=Mus musculus GN=Hbb-b1 PE=1 SV=2	Y	
HNRH1_MOUSE	Heterogeneous nuclear ribonucleoprotein H OS=Mus musculus GN=Hnrnph1 PE=1 SV=3		Y
HCDH_MOUSE	Hydroxyacyl-coenzyme A dehydrogenase, mitochondrial OS=Mus musculus GN=Hadh PE=1 SV=2		Y
IL17F_MOUSE	Interleukin-17F OS=Mus musculus GN=Il17f PE=2 SV=1	Y	
K1KB1_MOUSE	Kallikrein 1-related peptidase b1 OS=Mus musculus GN=Klk1b1 PE=2 SV=1	Y	
K1B11_MOUSE	Kallikrein 1-related peptidase b11 OS=Mus musculus GN=Klk1b11 PE=2 SV=1	Y	
K1B16_MOUSE	Kallikrein 1-related peptidase b16 OS=Mus musculus GN=Klk1b16 PE=1 SV=2	Y	
K1B21_MOUSE	Kallikrein 1-related peptidase b21 OS=Mus musculus GN=Klk1b21 PE=2 SV=3	Y	
K1B22_MOUSE	Kallikrein 1-related peptidase b22 OS=Mus musculus GN=Klk1b22 PE=1 SV=1	Y	
K1B24_MOUSE	Kallikrein 1-related peptidase b24 OS=Mus musculus GN=Klk1b24 PE=2 SV=3	Y	
K1B26_MOUSE	Kallikrein 1-related peptidase b26 OS=Mus musculus GN=Klk1b26 PE=2 SV=1	Y	
K1B27_MOUSE	Kallikrein 1-related peptidase b27 OS=Mus musculus GN=Klk1b27 PE=1 SV=1	Y	
K1KB3_MOUSE	Kallikrein 1-related peptidase b3 OS=Mus musculus GN=Klk1b3 PE=1 SV=1	Y	
K1KB5_MOUSE	Kallikrein 1-related peptidase b5 OS=Mus musculus GN=Klk1b5 PE=2 SV=1	Y	
K1KB8_MOUSE	Kallikrein 1-related peptidase b8 OS=Mus musculus GN=Klk1b8 PE=2 SV=1	Y	
K1KB4_MOUSE	Kallikrein 1-related peptidase-like b4 OS=Mus musculus GN=Klk1b4 PE=1 SV=1	Y	
KLK1_MOUSE	Kallikrein-1 OS=Mus musculus GN=Klk1 PE=1 SV=3	Y	Y
K1C18_MOUSE	Keratin, type I cytoskeletal 18 OS=Mus musculus GN=Krt18 PE=1 SV=5		Y
K1C19_MOUSE	Keratin, type I cytoskeletal 19 OS=Mus musculus GN=Krt19 PE=1 SV=1		Y
K2C8_MOUSE	Keratin, type II cytoskeletal 8 OS=Mus musculus GN=Krt8 PE=1 SV=4		Y
NDUAA_MOUSE	NADH dehydrogenase [ubiquinone] 1 alpha subcomplex subunit 10, mitochondrial OS=Mus musculus GN=Ndufa10 PE=1 SV=1		Y
AMYP_MOUSE	Pancreatic alpha-amylase OS=Mus musculus GN=Amy2 PE=1 SV=2		Y
PPIA_MOUSE	Peptidyl-prolyl cis-trans isomerase A OS=Mus musculus GN=Ppia PE=1 SV=2	Y	
PPIB_MOUSE	Peptidyl-prolyl cis-trans isomerase B OS=Mus musculus GN=Ppib PE=1 SV=2		Y
PRDX1_MOUSE	Peroxiredoxin-1 OS=Mus musculus GN=Prdx1 PE=1 SV=1		Y
PRDX2_MOUSE	Peroxiredoxin-2 OS=Mus musculus GN=Prdx2 PE=1 SV=3		Y
PRDX6_MOUSE	Peroxiredoxin-6 OS=Mus musculus GN=Prdx6 PE=1 SV=3		Y
PGAM1_MOUSE	Phosphoglycerate mutase 1 OS=Mus musculus GN=Pgam1 PE=1 SV=3		Y
PMM2_MOUSE	Phosphomannomutase 2 OS=Mus musculus GN=Pmm2 PE=1 SV=1		Y
PIP_MOUSE	Prolactin-inducible protein homolog OS=Mus musculus GN=Pip PE=2 SV=3	Y	
PDIA3_MOUSE	Protein disulfide-isomerase A3 OS=Mus musculus GN=Pdia3 PE=1 SV=2		Y
PDIA1_MOUSE	Protein disulfide-isomerase OS=Mus musculus GN=P4hb PE=1 SV=2		Y
KPYM_MOUSE	Pyruvate kinase PKM OS=Mus musculus GN=Pkm PE=1 SV=4		Y
RAB2A_MOUSE	Ras-related protein Rab-2A OS=Mus musculus GN=Rab2a PE=1 SV=1		Y
RENI1_MOUSE	Renin-1 OS=Mus musculus GN=Ren1 PE=1 SV=1	Y	
RENI2_MOUSE	Renin-2 OS=Mus musculus GN=Ren2 PE=1 SV=1	Y	
GDIR1_MOUSE	Rho GDP-dissociation inhibitor 1 OS=Mus musculus GN=Arhgdia PE=1 SV=3		Y
TRFE_MOUSE	Serotransferrin OS=Mus musculus GN=Tf PE=1 SV=1		Y
ALBU_MOUSE	Serum albumin OS=Mus musculus GN=Alb PE=1 SV=3		Y
SIAS_HUMAN	Sialic acid synthase OS=Homo sapiens GN=NANS PE=1 SV=2		Y
SRGP3_MOUSE	SLIT-ROBO Rho GTPase-activating protein 3 OS=Mus musculus GN=Srgap3 PE=1 SV=1	Y	
SNX6_MOUSE	Sorting nexin-6 OS=Mus musculus GN=Snx6 PE=1 SV=2		Y
SODM_MOUSE	Superoxide dismutase [Mn], mitochondrial OS=Mus musculus GN=Sod2 PE=1 SV=3		Y
PRDX3_MOUSE	Thioredoxin-dependent peroxide reductase, mitochondrial OS=Mus musculus GN=Prdx3 PE=1 SV=1		Y
TCEA1_MOUSE	Transcription elongation factor A protein 1 OS=Mus musculus GN=Tcea1 PE=1 SV=2	Y	
TAGL_MOUSE	Transgelin OS=Mus musculus GN=Tagln PE=1 SV=3		Y
TKT_MOUSE	Transketolase OS=Mus musculus GN=Tkt PE=1 SV=1		Y
TPIS_MOUSE	Triosephosphate isomerase OS=Mus musculus GN=Tpi1 PE=1 SV=4	Y	
KCY_MOUSE	UMP-CMP kinase OS=Mus musculus GN=Cmpk1 PE=1 SV=1		Y
MYO1D_MOUSE	Unconventional myosin-Id OS=Mus musculus GN=Myo1d PE=1 SV=1	Y	
VDAC2_MOUSE	Voltage-dependent anion-selective channel protein 2 OS=Mus musculus GN=Vdac2 PE=1 SV=2	Y	
